# Expression analysis of flavonoid biosynthesis genes during *Arabidopsis thaliana *silique and seed development with a primary focus on the proanthocyanidin biosynthetic pathway

**DOI:** 10.1186/1756-0500-3-255

**Published:** 2010-10-07

**Authors:** Christiane Katja Kleindt, Ralf Stracke, Frank Mehrtens, Bernd Weisshaar

**Affiliations:** 1 Bielefeld University, Department of Biology, Genome Research, 33594 Bielefeld, Germany; 2 International NRW Graduate School in Bioinformatics and Genome Research, Bielefeld University, 33594 Bielefeld, Germany

## Abstract

**Background:**

The coordinated activity of different flavonoid biosynthesis genes in *Arabidopsis thaliana *results in tissue-specific accumulation of flavonols, anthocyanins and proanthocyanidins (PAs). These compounds possess diverse functions in plants including light-attenuation and oxidative stress protection. Flavonoids accumulate in a stimulus- and/or development-dependent manner in specific parts of the plant. PAs accumulate in the seed coat (testa).

**Findings:**

We describe the biological material and the preparation of total RNA for the AtGenExpress developmental silique and seed series. AtGenExpress ATH1 GeneChip expression data from the different stages were reanalyzed and verified using quantitative real time PCR (qPCR). We observed organ-specific transcript accumulation of specific flavonoid biosynthetic genes consistent with previously published data and our PA compound accumulation data. In addition, we investigated the regulation of PA accumulation in developing *A. thaliana *seeds by correlating gene expression patterns of specific flavonoid biosynthesis genes with different seed embryonic developmental stages and organs and present two useful marker genes for isolated valve and replum organs, as well as one seed-specific marker.

**Conclusions:**

Potential caveats of array-based expression data are discussed based on comparisons with qPCR data. Results from ATH1 microarray and qPCR experiments revealed a shift in gene activity from general flavonoid biosynthesis at early stages of seed development to PA synthesis at late (mature) stages of embryogenesis. The examined PA accumulation-associated genes, including biosynthetic and regulatory genes, were found to be exclusively expressed in immature seeds. Accumulation of PAs initiates at the early heart stage of silique and seed development. Our findings provide new insights for further studies targeting the PA pathway in seeds.

## Background

Flavonoids are aromatic amino acid-derived secondary metabolites with a multitude of roles in plants. For example, they act as antimicrobial agents, light protection pigments, feeding deterrents against pathogens and herbivores [1] and are, among other pigments, crucial for flower coloration, attracting insects for pollination and seed dispersal [2]. Flavonoids are subdivided into several classes, with three major ones in *Arabidopsis thaliana*: flavonols, anthocyanins, and proanthocyanidins (PAs, condensed tannins) [reviewed in [3]]. Flavonols contribute to UV-protection [4,5], while the red and purple anthocyanin pigments play a role in light-attenuation and serve as antioxidants [reviewed in [6]]. PAs are also effective antioxidants [7], and, in *A. thaliana*, responsible for the brown color of seed coats (testa) [reviewed in [3]] subsequent to their oxidation [8,9].

Mature seeds consist of the embryo, the end product of embryogenesis, surrounded by the seed coat [reviewed in [10]]. During embryogenesis, the fertilized egg cell (zygote) divides and differentiates into the following main stages: four cell stage, globular stage, heart stage, torpedo stage, walking-stick stage, curled cotyledon stage, and green cotyledon stage [[11], reviewed in [12]]. Recent studies have shown that silique and seed development are highly coordinated processes which involve embryogenesis and endosperm development, as well as the maternal development of the seed coat and siliques hulls [13,14]. The testa of *A. thaliana *seeds mainly consist of the inner and outer integument. PAs exclusively accumulate in the innermost inner integument (the endothelium cell layer), the micropylar area and the chalazal bulb of *A. thaliana *seeds [9,15,16]. Mutants defective in PA accumulation, referred to as *transparent testa *(*tt *[17,18]), have yellow to pale brown colored seeds [15].

**Figure 1:**
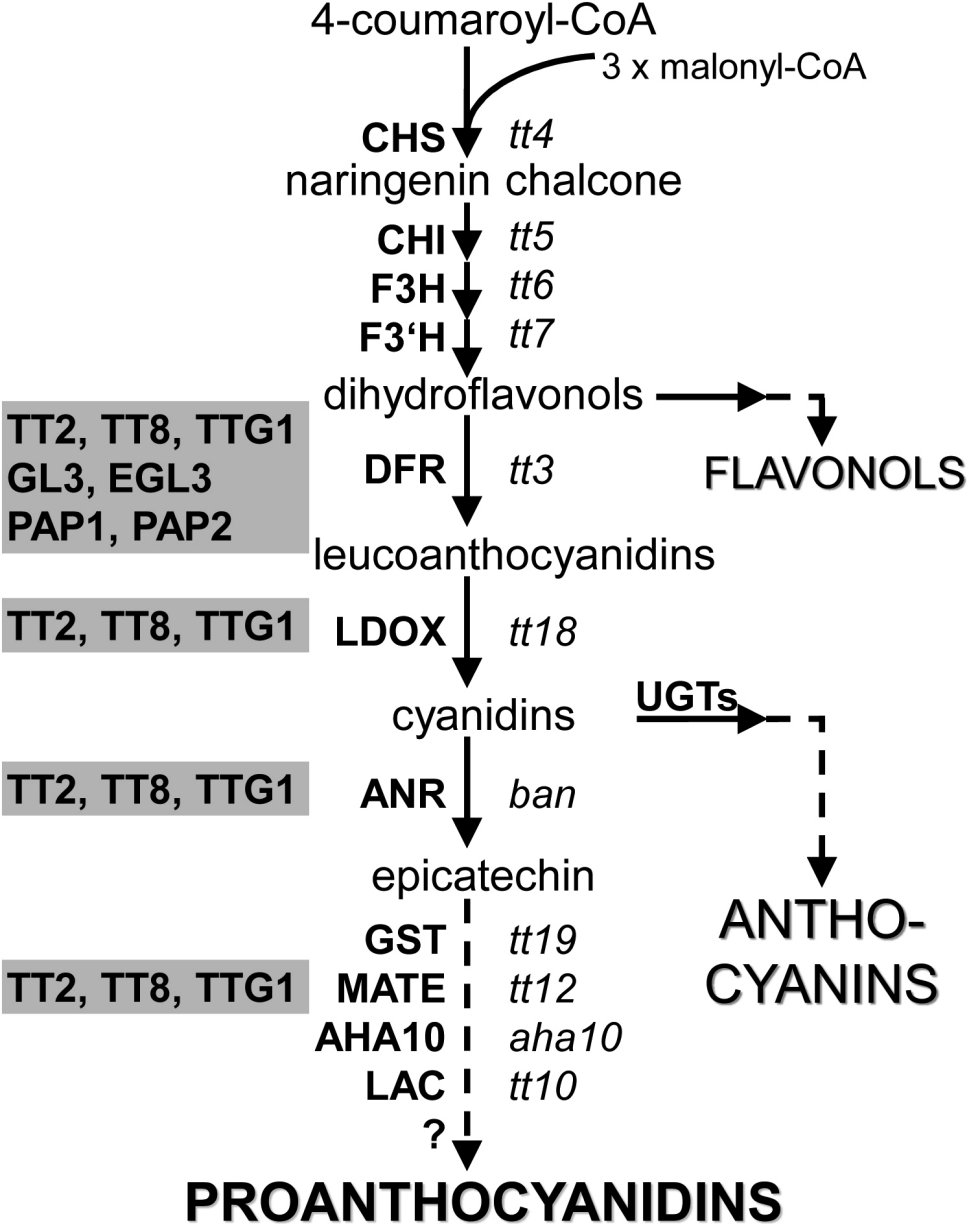
**Simplified, schematic representation of flavonoid biosynthesis in *A. thaliana *with a focus on proanthocyanidins**. Essential steps of the flavonoid biosyntheses leading to proanthocyanidins (PAs) in the seed are presented. Enzymes and transcriptional regulators (grey shaded) are indicated by bold, capital letters; corresponding mutant plant lines are in *italics*. Dashed arrows tag multiple steps which are not fully understood. The question mark indicates a limited knowledge of this biosynthetic step and a restricted understanding of the involvement of the associated factors. Abbreviations are as follows: *ANR/BAN *anthocyanidin reductase, *AHA10 *P-type H^+^-ATPase, *CHS *chalcone synthase, *CHI *chalcone isomerase, *DFR *dihydroflavonol 4-reductase, *EGL3 *enhancer of glabra3, *F3 H *flavanone 3β-hydroxylase, *F3'H *flavonoid 3'-hydroxylase, *GL3 *glabra3, *GST *glutathione S transferase, *LAC *laccase-like, *LDOX *leucoanthocyanidin dioxygenase, *MATE *multidrug and toxic compound extrusion-type transporter, *PAP *production of anthocyanin pigments, *TT *transparent testa, *TTG *transparent testa glabra, *UGTs *UDP dependent glycosyltransferases. Modified from [1,67,68].

Figure 1 presents a simplified, schematic representation of the flavonoid biosynthesis pathway in *A. thaliana *with a main focus on PA biosynthesis. These metabolites are synthesized in a series of enzymatic steps beginning with chalcone synthase (CHS), which synthesizes naringenin chalcone from 4-coumaroyl-CoA and malonyl-CoAs. Activities of the enzymes chalcone isomerase (CHI), flavanone 3-hydroxylase (F3H), and flavonoid 3'-hydroxylase (F3'H) result in the synthesis of dihydroflavonols. The enzyme dihydroflavonol 4-reductase (DFR) reduces dihydroflavonols to leucoanthocyanidins which are in turn converted to anthocyanidins by the activity of leucoanthocyanidin dioxygenase (LDOX). Anthocyanidins form a branching point in the flavonoid biosynthesis synthesizing anthocyanins (via glycosylation by UDP-dependent glucosyltransferases (UGTs)) or epicatechin (by the enzyme anthocyanidin reductase (ANR; BANYULS, BAN [19])). Epicatechin is the precursor of PAs [19], formed via the involvement of different enzymes including glutathione S transferase (GST), a multidrug and toxic compound extrusion-type transporter (MATE), a P-type H^+^-ATPase (AHA10), and a laccase-like (LAC). Genes encoding these proteins are collectively referred to as PA biosynthetic genes, although, for instance, the transporter is relevant for localization and not synthesis. Steps in late PA synthesis have yet to be elucidated [20].

The expression of flavonoid biosynthesis genes is regulated by a hierarchy of different transcription factors (TFs), as reviewed in Lepiniec *et al. *[3]. The accumulation of PAs and anthocyanin compounds in *A. thaliana *is mainly regulated at the level of transcription by a heterotrimeric complex, composed of the WD40 protein TRANSPARENT TESTA GLABRA1 (TTG1), a basic helix-loop-helix (bHLH), and a R2R3-MYB (MYB) TF, which interacts with target promoters of flavonoid biosynthesis genes [reviewed in [21]]. In *A. thaliana *seeds, PA biosynthesis is known to be under the control of TTG1, TT8 (bHLH42), and TT2 (MYB123) [22-24]. In addition, the zinc-finger protein TT1 has been shown to be involved in seed-specific PA accumulation [25]. Anthocyanin biosynthesis is mainly regulated by TTG1, which interacts with several bHLHs (GLABRA3 (GL3), ENHANCER OF GLABRA3 (EGL3), TT8) and the R2R3-MYBs (PRODUCTION OF ANTHOCYANIN PIGMENT1 (PAP1, MYB75), PAP2 (MYB90), MYB113, and MYB114) [26].

Flavonoid accumulation has been widely used to study gene regulation in plants due to tissue-specific regulation of flavonoid biosynthesis genes in response to a variety of stress conditions [reviewed in [27]], as well as during development [reviewed in [28]]. Here, we present expression profiles of flavonoid biosynthesis genes during silique and seed development in *A. thaliana*. We relate expression data of flavonoid-, and PA biosynthesis genes in particular, with specific seed developmental stages. Finally, our organ-specific transcript data (from silique hulls and isolated seeds) provides useful markers for tissue-specific gene expression studies.

## Results and Discussion

### Correlation of silique length to embryo developmental stages and timing of proanthocyanidin accumulation in the testa during seed development

**Figure 2:**
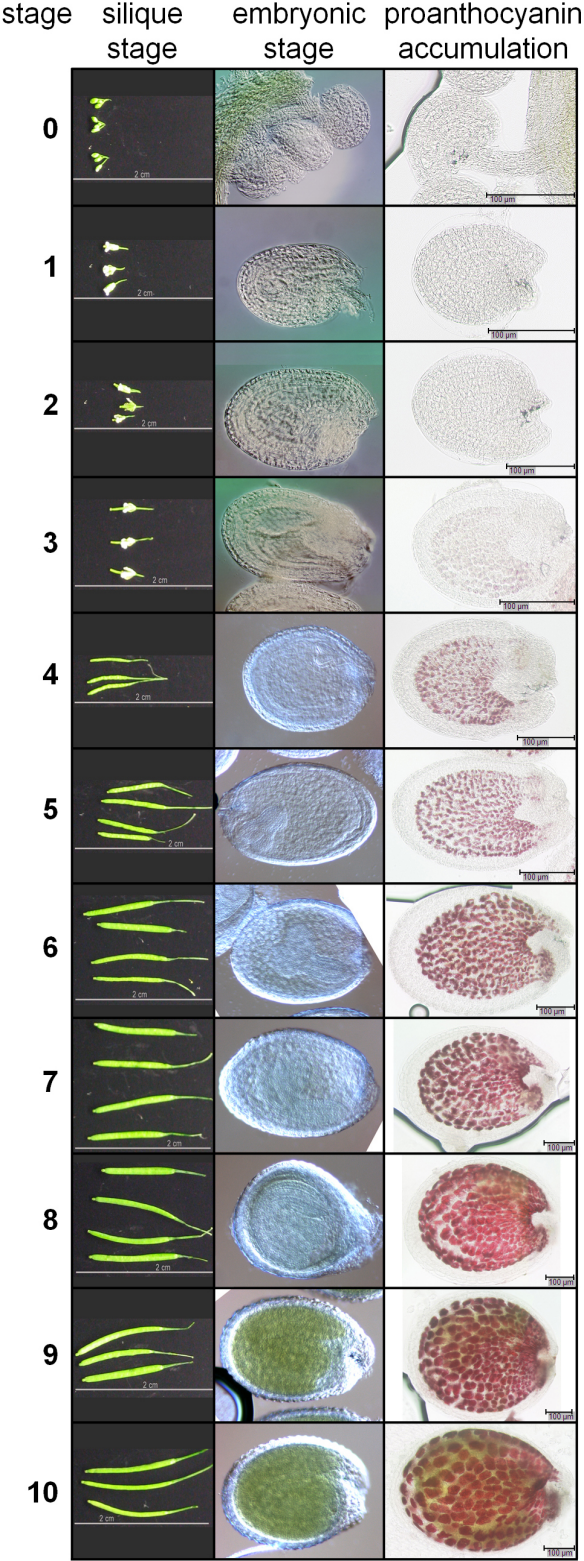
**Developmental stages of *A. thaliana *siliques and single seeds, and accumulation of proanthocyanidins**. Developmental stages of *A. thaliana *siliques are correlated to embryonic stages of single seeds according to Bowman [11], and to the accumulation of proanthocyanidins in the seed coat using vanillin staining.

Silique development was followed using light microscopy, focusing on stages of embryo development (according to Bowman [11]) in enclosed immature seeds. Figure 2 shows the correlation of the different stages of embryogenesis (listed in Table 1) to silique length at defined growth conditions, as well as to PA accumulation during seed development of wild type *A. thaliana *Col-0. The accumulation of PAs in the seed coat endothelium was visualized using vanillin staining.

**Table 1:** Definition of stages for *A. thaliana *seed and silique development used in this work.

stage	stages of embryogenesis	hours after flowering	stage length^1 ^[mm]	sample harvested for ATH1 GeneChip hybridization	**AtGen-Express accession (ATGE) No**.
0	mature embryosac	0	1.2-1.4	**-**	**-**
1	up to four cells	12-24	2.0-2.2	**-**	**-**
2	early globular to mid globular	24-48	2.4-2.8	**-**	**-**
3	mid globular to early heart	48-66	3.8-4.2	siliques containing seeds	ATGE_76
4	early heart to late heart	66-84	6.0-6.8	siliques containing seeds	ATGE_77
5	late heart to mid torpedo	84-90	8.0-9.2	siliques containing seeds	ATGE_78
6	mid torpedo to late torpedo	90-96	10.0-11.2	isolated seeds	ATGE_79
7	late torpedo to early walking-stick	96-108	11.6-12.2	isolated seeds	ATGE_81
8	walking-stick to early curled cotyledons	108-120	12.4- 13.0	isolated seeds	ATGE_82
9	curled cotyledons to early green cotyledons	120-144	12.8-13.6	isolated seeds	ATGE_83
10	green cotyledons	144-192	13.6-14.4	isolated seeds	ATGE_84

^1^Minimal and maximal flower and silique length observed under our green house conditions.

PA accumulation was first detected at developmental stage 3, corresponding to mid globular to early heart stage (Figure 2) and continued until the embryo turned green (stages 9-10). The observed timing of PA accumulation during seed development in *A. thaliana *Col-0 (Figure 2) is consistent with that noted by Debeaujon *et al. *[16] in the *Wassilewskija *accession.

### Verification of silique and seed developmental series ATH1 GeneChip data by quantitative real time PCR

As part of the AtGenExpress expression atlas, RNA from eight developmental stages (stages 3-10 with siliques containing seeds of the stages 3-5 and isolated seeds of the stages 6-10; see Table 1 and Methods) were used for ATH1 GeneChip experiments [29]. We reproduced the biological samples used for the AtGenExpress developmental series (see Methods) for quantitative real time PCR (qPCR) analyses. The siliques containing seeds and isolated seed samples provide a limited resolution of silique and seed developmental gene expression. Therefore, to increase resolution and determine organ specificity of gene expression, we included samples from earlier stages of silique development (0-2; Table 1), and from different parts of developing siliques (stages 6-10; Figure 2), such as isolated seeds and isolated silique hulls consisting of valves and replum organs.

We checked for reproducibility and validity of the developmental series data using qPCR, which is generally accepted as the standard [30,31] and more sensitive method [32,33] for verification of microarray gene expression data. In general, qPCR results verified the array results. However, in some cases, differences in expression were observed between the two techniques, reflecting the limitations of using arrays for gene expression studies. These discrepancies may be due to: i) variations in biological samples, ii) hybridization-based sequence specific effects [34], and/or iii) signal saturation during ATH1 hybridization. For example, expression of the *CHS *gene is controlled by several different stimuli [reviewed in [3]] acting on the biological samples. Therefore, the ATH1 GeneChip expression profile of *CHS *is probably difficult to verify by qPCR.

In addition, cross hybridization can occur within multiple genes resulting in nonspecific gene expression profiles [35]. We found that the ATH1 gene expression profile of *LDOX *represented transcripts of two related *A. thaliana *genes (*leucoanthocyanidin dioxygenase*, putative (AT4G22870); *LDOX *(AT4G22880)). However, qPCR expression data specifically represented only the LDOX gene. Finally, signal saturation during ATH1 hybridization may also prevent accurate verification of gene expression by qPCR [36,37]. This was observed for *ARABIDOPSIS THALIANA SEED GENE3 (ATS3) *and *TT10*. Although qPCR results for these two genes did not match the array results, they were consistent with previously published data.

A correlation analysis of genes showing a non saturated, gene-specific ATH1 expression profile reveals a good linear correlation (*r *= 0.52) of ATH1 GeneChip expression data with qPCR transcript levels (log_2 _transformed) using the Pearson product-moment correlation coefficient (PMCC, *r*).

### Evaluation of specificity of isolated seed, valve and replum organs by organ-specific expression analysis

**Figure 3:**
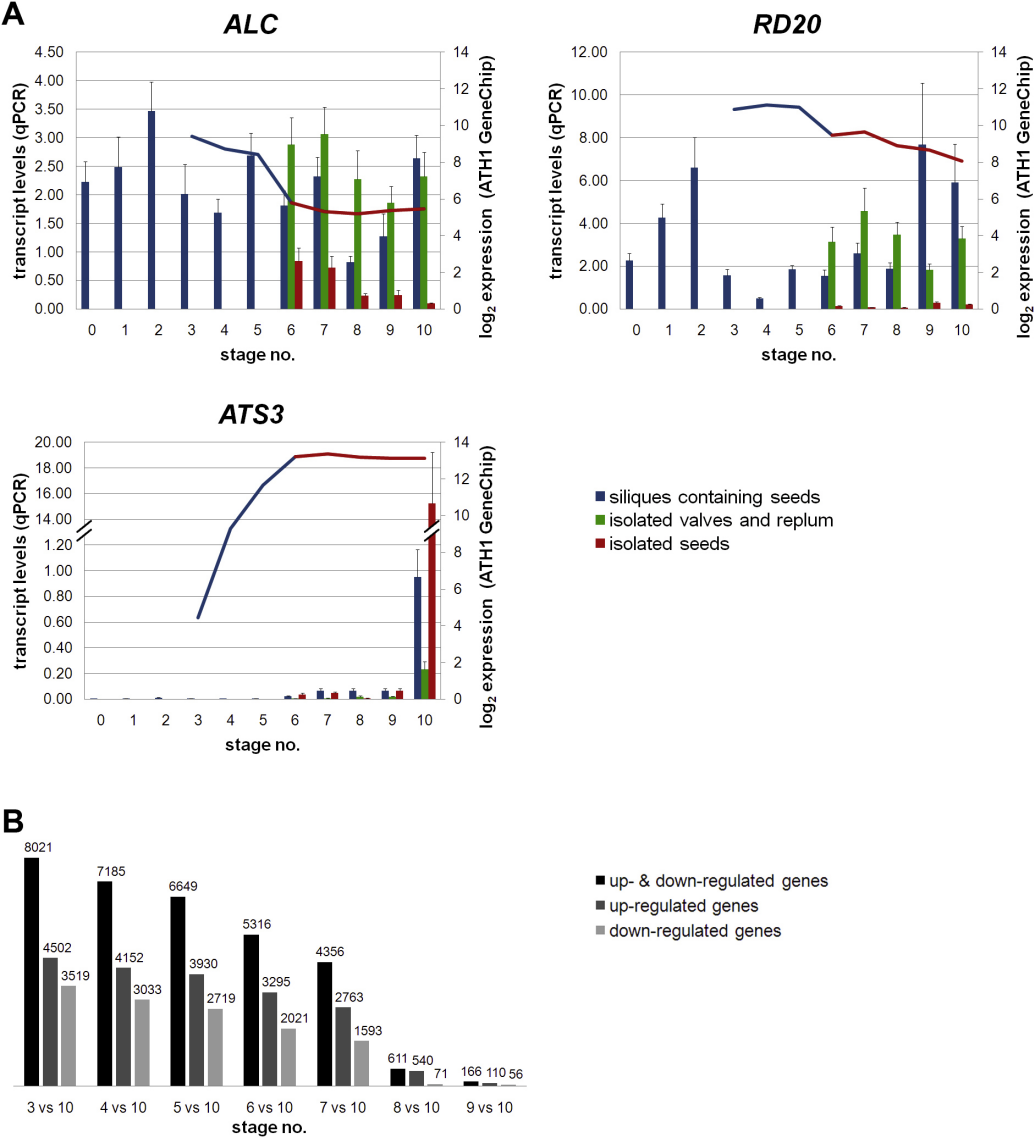
**Organ-specific expression of selected marker genes**. **(A) **qPCR transcript levels are presented as bars (relative expression levels shown on the primary axis were normalized to *TIM44*-*2*), while corresponding log_2 _expression values of ATH1 GeneChips are displayed as curves on the secondary axis. *ALC *is expressed in the valves margins of the silique [39]. *RD20 *is expressed specifically in siliques, but not in seeds [40]. *ATS3 *expression is seed-specific [38]. The presented genes are useful markers for separated *A. thaliana *silique samples (isolated valve and replum organs, isolated seeds). **(B) **ATH1 GeneChip hybridization data were used to determine the number of differentially regulated genes at each developmental stage normalized to stage 10. The number of significantly up- and down-regulated genes is shown (log_2 _*foldchange *≥1.0 or log_2 _*foldchange*≤-1.0, *p *≤ 0.05). Color key: blue, siliques containing seeds; green, isolated valves and replum organs; red, isolated seeds. Abbreviations are as follows: *ALC *alcatraz, *RD20 *responsive to dehydration 20, *ATS3 A. thaliana *seed gene3, *TIM44*-*2 *translocase inner membrane subunit 44-2.

To evaluate organ specificity of the isolated samples, we selected two genes with known valve and replum-specific expression (*ALCATRAZ, ALC *and *RESPONSIVE TO DEHYDRATION20, RD20*) and one with a seed-specific expression pattern (*ATS3*) [38]. *ALC *is expressed in valve margins of the silique, as shown by *GUS *promoter studies [39] and *RD20 *transcripts are present in siliques but not in seeds, as shown by Northern blot analysis [40]. In our study, expression of *ALC *and *RD20 *genes was typically higher in isolated valve and replum organs compared to silique containing seeds and very low in isolated seed samples (Figure 3A). Low transcript levels were detected in isolated seed sample leading back to sample separation method. In contrast, a late seed-specific expression was observed for *ATS3 *(Figure 3A).

Although separation of small siliques (stages 6-8; Figure 2) into seeds, valves and replum is difficult with immature seeds sticking to the replum, we were able to detect organ-specific gene expression patterns using qPCR and confirmed that our separation procedure was successful since minimal variations in corrected crossing points (cCPs) were observed for *ALC*, *RD20*, and *ATS3 *(Additional file 1). This was not previously resolved in the ATH1 GeneChip data set. Moreover, these three genes serve as useful markers for isolated seed (*ATS3*), and valve/replum organs (*ALC, RD20*).

### Reanalysis of the ATH1 silique and seed developmental series reveals stage-specific expression patterns of flavonoid biosynthesis genes

We evaluated embryo developmental stage-specific expression profiles using the previously generated ATH1 GeneChip dataset [29]. A heat map analysis of tissue specific marker genes, principle component analysis of the seed developmental samples, as well as developmental analyses of the ATH1 GeneChips, which show two main, opposing expression trends from early to late seed stages, have been presented and discussed in Schmid *et al. *[29].

First, differentially expressed genes were obtained (log_2 _*foldchange *≥1.0 or log_2 _*foldchange*≤-1.0, *p *≤ 0.05) for each developmental stage by comparing expression at each stage to that at stage 10 (Figure 3B). Second, MapMan [41] was used to identify enriched functional processes at each stage. The highest number of differentially expressed genes was detected at mid globular to early heart stage (stage 3) with 8021 genes exhibiting significantly altered expression at this stage. These results were highly similar to the AtGenExpress expression atlas data [29].

We also found that expression patterns of flavonoid biosynthesis genes were dramatically altered, with a majority of these genes down-regulated during silique and seed development in the ATH1 GeneChip datasets (data not shown).

In addition, gene expression data was consistent with the onset of PA accumulation in the seed coat at the early heart stage, detected using vanillin staining (Figure 2). Similar results were reported in an ATH1 expression profile study conducted on developmental stages 1-3 in the Landsberg *erecta *accession [13] showing a continuous activation of the PA branch. Our analysis also shows that PA accumulation does not occur at early developmental stages (0-2, Table 1).

Using qPCR, we also examined the gene expression profile of the flavonoid biosynthesis regulator *TT1 *that is not represented on the ATH1 chip. We found that *TT1 *transcripts are exclusively present in developing young seeds (data not shown), similar to that reported by Sagasser *et al. *[25].

### Expression of flavonoid biosynthesis genes is seed-specific

**Figure 4:**
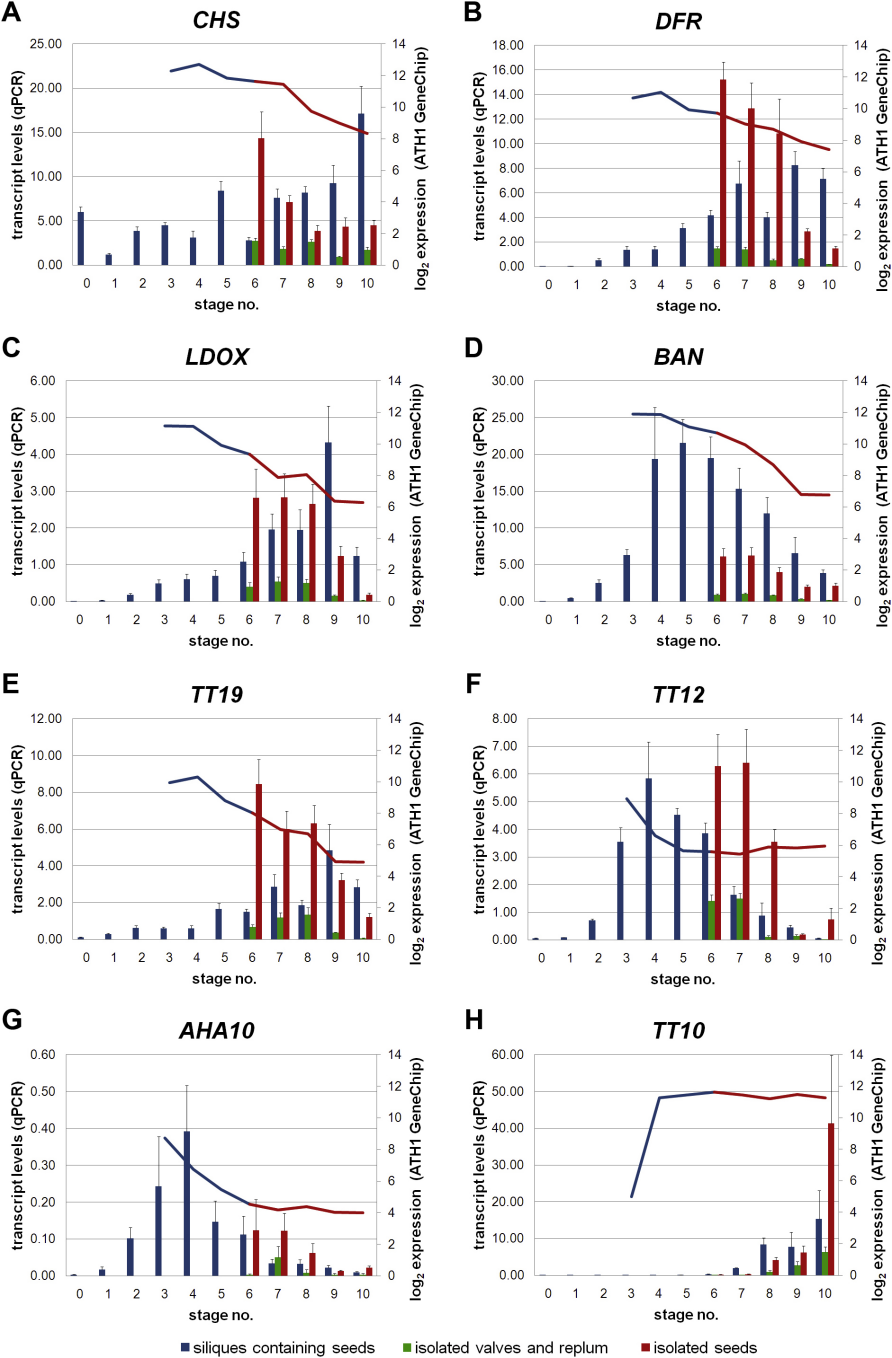
**Expression patterns of selected flavonoid biosynthesis genes during silique and seed development in *A. thaliana***. qPCR analyses were performed to verify ATH1 GeneChip expression values. Relative transcript levels (bars, primary axis; normalized to *TIM44*-*2*) correlate to log_2 _expression values of ATH1 GeneChips (curve, secondary axis). **(A**-**C) **Seed specific expression of structural flavonoid biosynthesis genes is shown. **(D**-**H) **Seed specific expression patterns of proanthocyanidin biosynthesis genes are presented. Color key: blue, siliques containing seeds; green, isolated valves and replum organs; red, isolated seeds. Abbreviations are as follows: *AHA10 *P-type H^+^-ATPase, *BAN *anthocyanidin reductase, *CHS *chalcone synthase, *DFR *dihydroflavonol 4-reductase, *LDOX *leucoanthocyanidin dioxygenase, *TIM44*-*2 *translocase inner membrane subunit 44-2, *TT *transparent testa, *TT10 *laccase-like, *TT12 *multidrug and toxic compound extrusion-type transporter, *TT19 *glutathione S transferase.

We also examined the organ-specific expression patterns of flavonoid biosynthesis genes during silique and seed development using qPCR. The flavonoid biosynthetic genes *CHS*, *DFR*, *LDOX*, and *BAN *(Figure 4A-D) were expressed in isolated seeds consistent with the ATH1 GeneChip isolated seeds expression data. Likewise, the PA biosynthesis-specific genes *TT19*, *TT12*, *AHA10*, and *TT10 *(Figure 4E-H) exhibit isolated seed-specific expression [16]. Seed-specific expression of *TT10 *has been previously reported using semi-quantitative reverse transcription PCR (RT-PCR) by Pourcel *et al. *[42]. Although ATH1 and qPCR results do not account for expression differences within different parts of the seed, it is conceivable that the detected seed-specific expression of PA biosynthetic genes is due to expression in the testa where PAs are primarily known to accumulate [16]. Only basal transcript levels of these genes were detected in isolated valves and replum organs suggesting that PA accumulation-associated genes are not expressed in silique hulls.

### *TT2 *and *TT8 *show organ-specific gene expression in isolated seeds

During *A. thaliana *seed development, PAs accumulate specifically in the endothelium layer of the seed coat [8,43] via the activity of at least four regulatory TFs: *TT2*, *TT8*, *TTG1*, and *TT16 *[23,44]. These TFs are required for the expression of several flavonoid biosynthesis genes in maturing seeds [22,23], such as *DFR *and *BAN *(Figure 1, 4B, D).

**Figure 5:**
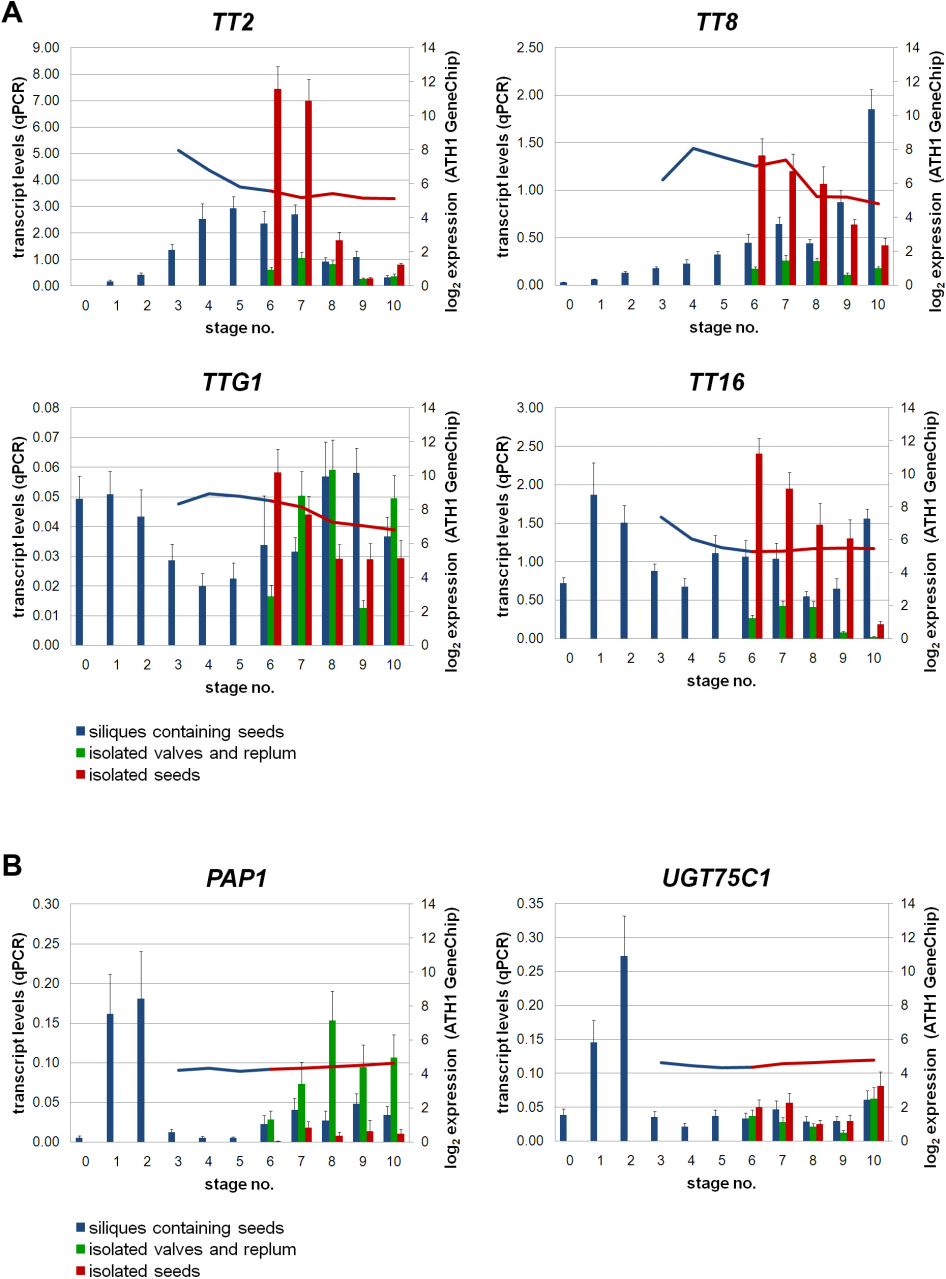
**Expression patterns of selected proanthocyanidin and anthocyanin accumulation genes during silique and seed development**. qPCR analyses were performed to verify ATH1 GeneChip expression values. Relative transcript levels (bars, primary axis; normalized to *TIM44*-*2*) correlate to log_2 _expression values of ATH1 GeneChips (curve, secondary axis). **(A) **Expression patterns of regulatory genes responsible for proanthocyanidin accumulation are shown during *A. thaliana *silique and seed development. **(B) **Anthocyanin accumulation-associated genes show global faint gene expression. *PAP1 *and *UGT75C1*, which is known to be regulated by PAP1 are significantly up-regulated at early developmental stages. Color key: blue, siliques containing seeds; green, isolated valves and replum organs; red, isolated seeds. Abbreviations are as follows: *PAP *production of anthocyanin pigments, *TIM44*-*2 *translocase inner membrane subunit 44-2, *TT *transparent testa, *TTG *transparent testa glabra, *UGT75C1 *UDP dependent glycosyltransferase.

We studied gene expression profiles of *TT2*, *TT8*, *TTG1*, and *TT16 *during silique and seed development (Figure 5A). *TT2 *is specifically expressed during early and mid developmental stages in siliques containing seeds (stages 4-7; Table 1), as previously reported by Nesi *et al. *[23]. Highest *TT2 *expression was observed in isolated seeds reflecting an activation of flavonoid biosynthesis towards PA accumulation. Expression of *TT8 *also appears to be isolated seed-specific, whereas *TTG1 *exhibits low level gene expression in all organs. The seed-specific expression of these genes reflects well the known regulation of PA biosynthesis by the heterotrimeric TTG1-TT8-TT2 complex [reviewed in [3]].

TT8 and TTG1 are also involved in the regulation of anthocyanin accumulation in vegetative tissues [22,45]. Since anthocyanin levels in the harvested plant material were below our detection limit, we concluded that the anthocyanin pathway was not activated (data not shown). This is supported by our qPCR results, since only very low transcript levels were detected for both genes in isolated valve and replum organs.

*TT16 *is expressed in immature siliques (stages 1 and 2), consistent with previously published RT-PCR results [44] and ATH1 expression profile results in the Landsberg *erecta *accession [13], and in isolated seeds. However, very low expression was detected in valves and replum organs.

### Genes involved in anthocyanin accumulation are activated during early stages of seed development

Although we could not measure detectable amounts of anthocyanins in the plant material harvested for qPCR experiments, we also looked at the expression profiles of anthocyanin biosynthesis genes that showed faint transcript levels in silique hulls (valves and replum organs). Figure 5B shows that gene expression levels of *PAP1 *and *UGT75C1 *are similar to that observed byATH1 GeneChips.

qPCR at earlier time points revealed a specific increase in *PAP1 *gene expression at developmental stages 1 and 2 (Figure 5B), consistent with ATH1 expression profile studies in the Landsberg *erecta *accession [13]. Likewise, *UGT75C1 *showed increased gene expression at early stages (Figure 5B), indicating a potential regulation by PAP1. This is in agreement with Tohge *et al. *[46] who reported an induction of *UGT75C1 *gene expression in *PAP1 *over-expressing plants.

In summary, we describe the biological material and the preparation of RNA samples for the AtGenExpress developmental silique and seed series. We successfully verified selected ATH1 GeneChip expression data using quantitative real time PCR (qPCR) on a biological replicate. We also included earlier developmental stages and addressed organ specificity (silique hulls, isolated seeds) by qPCR, both of which were not analyzable in the ATH1 GeneChip data. We present two useful marker genes for isolated valve and replum organs, as well as one seed-specific marker showing significant organ specific differences in expression.

We reanalyzed the ATH1 GeneChip silique and seed developmental series and found that expression levels of flavonoid biosynthesis genes are dramatically altered during development. Similar to the ATH1 GeneChip data, our qPCR results showed that PA biosynthesis-associated gene expression increases specifically in maturing seeds. Our data not only supports previous results on PA accumulation, but also shows that accumulation of PAs initiates at the early heart stage of silique and seed development providing a key finding for future studies targeted at investigating the PA pathway in seeds.

## Methods

### Plant growth conditions

For ATH1 GeneChip experiments, *Arabidopsis thaliana *seeds (Columbia-0 accession; Col-0) were sown on a peat-based substrate (70% ground white peat; 20% vermiculite 3-6 mm; 10% washed sand; 100 g/m^3 ^iron chelate; 100 g/m^3 ^Radigen^®^ trace elements), stratified in the dark for 3 days at 4°C and then transferred to the greenhouse. Plants were grown under short day conditions (8 h light/16 h dark cycles) for 3 weeks and then long day conditions (16 h light/8 h dark cycles) with additional illumination using SIL-lamps (Na- and Hg-steam, 180-200 μE) with 50-60% humidity and mean temperature of 18°C. Plants were harvested 8 weeks after germination.

For qPCR experiments, *A. thaliana *Col-0 plants were grown as described above with minor modifications. Plants were grown on peat-based substrate (70% white peat 0-8 mm, 20% vermiculite 3-6 mm, 10% sand, 1.4 g/L iron chelate, 2.1 g/L Radigen^® ^micronutrient fertilizer, pH5.5-6.0) with a mean temperature of 20°C.

### Harvest and stage confirmation of plant material for Affymetrix GeneChip experiments

To ensure harvest of homogenous samples, seed developmental stages were confirmed by light microscopy according to Bowman [11], as shown in Figure 2. Seeds originating from at least four different siliques per developmental stage were cleared prior to microscopy in a mixture of chloral hydrate/glycerol/water (8:2:1) for at least 2 h at room temperature. Microscopy was performed using a Nikon Eclipse E600 microscope system (Nikon GmbH, Düsseldorf, Germany). Batches of up to 100 mg of developing siliques (developmental stages 3-5 (see Table 1) or immature seeds isolated from opened siliques (developmental stages 6-10) were harvested on dry ice, transferred into liquid nitrogen and stored at -80°C until RNA isolation. For assignment to the various stages we focused on stages of embryo development (Figure 2) to ensure harvest of homogenous developmental stages. Anatomic terms for the description of plant organs were determined according to the Plant Ontology™Consortium [47].

### RNA extraction for Affymetrix GeneChip experiments

Extraction of total RNA was performed according to the protocol of Ruuska and Ohlrogge [48] with the following modifications: the extraction buffer contained water instead of tri-isopropylnaphtalene sulphonic acid, and the 2-butoxyethanol/isopropanol precipitation was performed only once. RNA resulting from this extraction procedure was purified using RNeasy Mini spin columns (Qiagen, Hilden, Germany) according to the manufacturer's protocol.

### Affymetrix GeneChip hybridization, data pre-processing and expression analyses

Total RNA extracted from eight developmental stages (siliques containing seeds, stages 3-5, and isolated seeds, stages 6-10; see Table 1) were used for Arabidopsis ATH1 GeneChip (Affymetrix UK Ltd., High Wycombe, UK) hybridizations. The labeling, hybridization, and array scanning were performed by Detlef Weigel's group (MPI of Developmental Biology, Tuebingen, Germany) according to Affymetrix's GeneChip Expression Analysis *Technical Manual *[49]. Microarrays were processed in triplicate for each developmental stage as part of the AtGenExpress expression atlas [29]. Table 1 presents the main stages of embryogenesis, the harvested sample organs, and the AtGenExpress accession number of the microarray datasets produced with the RNA samples described above. MIAME information for all samples, as well as microarray raw data are publicly available from AtGenExpress [29] at TAIR [50].

Microarray raw data of three biological replicates per developmental stage were analyzed using *EMMA 2 *[51] and the Bioconductor 2.4 software package [52]. Default settings were used for all Bioconductor modules. Expression values (log_2_) were extracted using robust multi-array analysis [53,54] and background correction was performed using perfect matches only which is implemented in the Bioconductor software package *affy *[55]. The linear model-fitting function in the *affyPLM *package [55] was used for quality assessment which was passed by all hybridized ATH1 GeneChips. One-way ANOVA (Bioconductor package *manova *[56]) was used to compute *p*-values of genes (*p *≤ 0.05). TAIR9 (May 2009 update) gene functional descriptions were employed [57]. MapMan (version 3.1.1 [41]) was used to identify functional processes that were altered statistically (*P *0.05) in the developmental gene sets.

### Harvest of plant material, RNA extraction and cDNA synthesis for qPCR experiments

The developmental stages of seeds were confirmed as described for Affymetrix GeneChip experiments. For plant harvest we concentrated on the main stages of embryogenesis and the silique length which are correlated in Table 1. Batches of up to 100 mg of flowers and developing siliques from stages 0-10 (see Table 1), and rosette leaves, were harvested on dry ice. Additionally, siliques from developmental stages 6-10 were opened and separated into isolated seeds and open siliques consisting of valves and replum. Plant material was immediately frozen in liquid nitrogen and stored at -80°C until RNA isolation. Total RNA extractions, including DNase treatment, were performed using the RNeasy Plant Mini Kit (Qiagen, Hilden, Germany) following the manufacturer's instructions. RNA concentration was quantified using a Nanodrop 1000 (Thermo Scientific, Wilmington, DE, USA). cDNA was synthesized from 2 μg of total RNA as previously described by Mehrtens *et al. *[58].

### qPCR and data analyses

Primers used for qPCR experiments were designed using ProbeFinder 2.45 (ROCHE Diagnostics-Applied Science, Mannheim, Germany) and are listed in additional file 2. qPCR was performed using the Platinum SYBR Green qPCR SuperMix UDG Kit (Invitrogen, Karlsruhe, Germany) following the manufacturer's instructions on a Rotor-Gene 6000 cycler (Qiagen, Hilden, Germany). For qPCR, cDNA from all biological samples were run in triplicate with cDNA from rosette leaves as the internal standard. Based on expression data from the AtGenExpress silique and seed developmental series [29] and Genevestigators' RefGenes tool (version V3 [59]), *A. thaliana TRANSLOCASE INNER MEMBRANE SUBUNIT 44*-*2 *(*TIM44*-*2 *[60]) was selected as the reference gene for qPCR normalization. *TIM44*-*2 *exhibited stable expression with minimal variation of the corrected crossing points (cCPs) across samples (Additional file 1) in pilot experiments and is therefore an excellent reference gene in accordance with MIQE guidelines [61]. Raw data was analyzed with the CAmpER software tool (Blom, personal communication [62]), which implements an algorithm based on a four parametric logistic model [63] of the fluorescence curve to calculate efficiency cCPs that were subsequently analyzed using the REST software [64]. Visualizations were created using Microsoft Office Excel 2007. Calculation of Pearson product-moment correlation coefficients was performed using Microsoft Office Excel 2007 according to Rodgers and Nicewander [65].

### Vanillin assay

Vanillin staining of intact seeds was performed according to Debeaujon *et al. *[15] to color monomers and terminal subunits of PAs, such as leucoanthocyanidins and catechins [66].

## Competing interests

The authors declare that they have no competing interests.

## Authors' contributions

CKK analyzed the ATH1 GeneChip experiment data, performed the qPCR experiments and the corresponding work, such as plant harvest, RNA isolation, and wrote the manuscript. FM produced and characterized the plant material and the RNA samples for ATH1 GeneChip hybridizations. RS and BW provided the application cases, supervised, and directed the whole project. All authors read and approved the final manuscript.

## Supplementary Material

Additional file 1 **Corrected crossing points of selected genes**. qPCR efficiency corrected crossing points of the reference gene and three selected genes to verify organ specificity. Click here for file

Additional file 2 **Primers used in qPCR**. Primer pairs (forward and reverse primer) for all genes used for qPCR [69]. Click here for file
