# Supplementary material for: Expression analysis of flavonoid biosynthesis genes during Arabidopsis thaliana silique and seed development with a primary focus on the proanthocyanidin biosynthetic pathway
Source: BMC Res Notes. 2010 Oct 7;3:255. doi: 10.1186/1756-0500-3-255 (PMC2958888; doi:10.1186/1756-0500-3-255)
Supplement: Additional file 1 — Corrected crossing points of selected genes. qPCR efficiency corrected crossing points of the reference gene and three selected genes to verify organ specificity. [file 1756-0500-3-255-S1.DOC]

### Additional file 1 – Corrected crossing points of selected genes.

*A. thaliana TRANSLOCASE INNER MEMBRANE SUBUNIT 44‑2* (*TIM44‑2* [60]) was used as the reference gene for quantitative real time PCR normalization. *ALCATRAZ* (*ALC* [39]), *RESPONSIVE TO DEHYDRATION 20* (*RD20* [40]), and the *A. thaliana SEED GENE 3* (*ATS3* [38]) were selected to verify organ specificity during seed development in *A. thaliana*. Corrected crossing points (cCPs) were normalized to an efficiency of 2.0 using the four parametric logistic model [63] implemented in CAmpER [62], **σ*_cCP_*** displays the standard deviation of cCPs.

| **stage** | **stage description** | ***TIM44‑2*** | | ***ALC*** | | ***RD20*** | | ***ATS3*** | |
| --- | --- | --- | --- | --- | --- | --- | --- | --- | --- |
|  |  | **cCP** | **σ_cCP_** | **cCP** | **σ_cCP_** | **cCP** | **σ_cCP_** | **cCP** | **σ_cCP_** |
| **0** | siliques containing seeds | 20.12 | 0.02 | 18.32 | 0.96 | 18.36 | 1.81 | 27.71 | 0.71 |
| **1** | siliques containing seeds | 18.19 | 0.11 | 17.58 | 0.62 | 16.93 | 0.45 | 28.76 | 0.92 |
| **2** | siliques containing seeds | 18.15 | 0.37 | 17.09 | 0.54 | 16.70 | 0.69 | 27.39 | 1.57 |
| **3** | siliques containing seeds | 17.43 | 0.46 | 18.26 | 0.80 | 18.27 | 0.78 | 28.02 | 1.64 |
| **4** | siliques containing seeds | 17.03 | 0.49 | 17.69 | 0.42 | 20.07 | 0.97 | 31.62 | 1.41 |
| **5** | siliques containing seeds | 19.80 | 0.18 | 18.90 | 0.28 | 18.40 | 0.19 | 29.32 | 1.14 |
| **6** | siliques containing seeds | 21.92 | 0.15 | 21.33 | 0.71 | 21.61 | 0.44 | 30.69 | 1.49 |
|  | isolated valves and replum | 18.69 | 0.24 | 18.38 | 0.51 | 17.87 | 0.74 | 29.39 | 1.36 |
|  | isolated seeds | 17.91 | 0.47 | 19.30 | 0.77 | 21.63 | 0.52 | 25.58 | 1.12 |
| **7** | siliques containing seeds | 19.19 | 0.60 | 19.41 | 0.22 | 17.68 | 0.76 | 25.46 | 0.97 |
|  | isolated valves and replum | 18.88 | 0.79 | 17.82 | 0.39 | 17.12 | 0.64 | 28.03 | 0.94 |
|  | isolated seeds | 17.48 | 0.44 | 19.81 | 0.19 | 21.91 | 0.99 | 24.90 | 1.25 |
| **8** | siliques containing seeds | 18.73 | 0.27 | 20.47 | 0.20 | 19.70 | 0.86 | 25.52 | 0.97 |
|  | isolated valves and replum | 20.02 | 0.03 | 19.82 | 0.59 | 18.41 | 0.32 | 28.26 | 1.97 |
|  | isolated seeds | 18.35 | 0.30 | 21.19 | 0.93 | 21.16 | 1.21 | 27.34 | 0.70 |
| **9** | siliques containing seeds | 19.79 | 0.59 | 19.02 | 0.94 | 16.33 | 0.10 | 26.06 | 0.73 |
|  | isolated valves and replum | 20.26 | 0.25 | 19.27 | 0.81 | 20.02 | 0.22 | 26.02 | 0.50 |
|  | isolated seeds | 20.37 | 0.29 | 22.60 | 0.78 | 25.04 | 0.50 | 23.23 | 0.36 |
| **10** | siliques containing seeds | 18.22 | 0.39 | 19.14 | 0.74 | 17.81 | 0.82 | 18.37 | 0.45 |
|  | isolated valves and replum | 18.32 | 0.21 | 18.33 | 0.70 | 17.08 | 0.26 | 19.43 | 0.89 |
|  | isolated seeds | 16.92 | 0.76 | 21.16 | 0.84 | 19.62 | 0.84 | 12.96 | 0.81 |
