# Supplementary material for: Expression analysis of flavonoid biosynthesis genes during Arabidopsis thaliana silique and seed development with a primary focus on the proanthocyanidin biosynthetic pathway
Source: BMC Res Notes. 2010 Oct 7;3:255. doi: 10.1186/1756-0500-3-255 (PMC2958888; doi:10.1186/1756-0500-3-255)
Supplement: Additional file 2 — Primers used in qPCR. Primer pairs (forward and reverse primer) for all genes used for qPCR [69]. [file 1756-0500-3-255-S2.DOC]

### Additional file 2 – Primers used in qPCR.

Primer pairs were designed using ProbeFinder 2.45 [69] and are presented below:

| **locus** | **forward primer** | | **reverse primer** | |
| --- | --- | --- | --- | --- |
|  | **ID** | **sequence (5' - 3')** | **ID** | **sequence (5' - 3')** |
| AT2G36070 | X005 | AAACAAGGTCAAGGCGTCTG | X006 | CACACTTGAAGAGACCTTTTTGG |
| AT5G67110 | X073 | GCAGCTTCAACTTCAAGTCCAGAC | X074 | GGTGGAACCTGTGGTAATCGCAT |
| AT2G33380 | X075 | TCAGCTACGTTACACTTCCGAGT | X076 | TGGCTTTGTGTATGTTGTCGAT |
| AT5G07190 | S783 | GCCGACGGTAACAAGGTG | S784 | TGACTTGGAATGTGTTCGTTG |
| AT5G13930 | K003 | CGCATCACCAACAGTGAACAC | K004 | TCCTCCGTCAGATGCATGTG |
| AT5G42800 | X025 | AACGGATGTGACGGTGTTTT | X026 | TCCATTCACTGTCGGCTTTA |
| AT1G61720 | G121 | AAGAAAACTGGACTGACGTTGAA | G122 | AACACCTTCGAGATTGGGTAAC |
| AT5G35550 | X013 | AAGGCAAATGGAGCACTCTC | X014 | CTACAGCTTTTGCCACACCTC |
| AT4G09820 | X015 | TGAATCAACCCATACGTTAGACA | X016 | GGGGTGTGACATGAGAAGTGT |
| AT5G24520 | O217 | GTCTTCTTCGCAGCCTGATT | O218 | AAACCAGCATGAAGTTTCCAA |
| AT1G34790 | K100 | GCAAATTCTCATAGGGTTCACTC | K101 | ATGTCCCCACATGTGCATC |
| AT5G41315 | X019 | AGTGTTTAGCCGTTCTCTTCTAGC | X020 | TGTCTTCCGTAATATGTTCTGTGG |
| AT1G17260 | K098 | TCCCGGGACTCTCCTGAT | K099 | GCATACACAGCAATTAATGTAGCC |
| At3g59030 | G119 | TGGTCTTCCTATTGGCTGTGT | G120 | CAATCATCCCCCACCAGAT |
| At5g17220 | S834 | GACAGGTAACAGCAGCTTGTCCA | S836 | CATCTTCTATGGCTGGAACTTGACCA |
| AT5G48100 | H448 | CAATGCATTGGCATGGTGTAGAG | H449 | CTCACATCCCTCTTCCACCAC |
| AT4G22880 | I040 | CGATGAAAAGATCCGTGAGAA | I041 | CACTCCCCAATCCAAAGATG |
| At1g56650 | X007 | AAATGGCACCAAGTTCCTGT | X008 | TCAGAGCTAAGTTTTCCTCTCTTGAT |
| AT1G63650 | X021 | TTGGCACGACCGAACATA | X022 | TTGATAGTCTGATCTTGTCGATATTGT |
| At4g14090 | O113 | GATCAGAGGAAGTGATCGAGGA | O114 | GCCAAAACAGCTGTCTGAGAA |
